# Editorial: Soil microbiome and agroecosystem multifunctionality

**DOI:** 10.3389/fmicb.2026.1914766

**Published:** 2026-07-13

**Authors:** Davey L. Jones, Emily C. Cooledge, Chengjie Ren, Tong Li, Ziting Wang

**Affiliations:** 1 School of Environmental and Natural Sciences, Bangor University, Bangor, United Kingdom; 2 College of Agriculture Sciences, Northwest A&F University, Yangling, China; 3 School of Agriculture and Biomanufacturing, Zhengzhou University, Zhengzhou, China; 4 State Key Lab for Conservation and Utilization of Subtropical Agri-Biological Resources, Guangxi Key Lab for Sugarcane Biology, College of Agriculture, Guangxi University, Nanning, China

**Keywords:** agricultural management, co-occurrence networks, ecosystem multifunctionality, microbial diversity, rhizosphere ecology, soil health, soil microbiome, sustainable agriculture

Soil microorganisms are responsible for the regulation of soil nutrient cycling and plant health, however, harnessing their power to promote agroecosystem resilience represents a major challenge for agriculture (Delgado-Baquerizo et al., 2016). Managing the microbiome, rather than working around it, offers a potential way to improve productivity while being less dependent on synthetic chemical inputs, more resilient to climate variability, more compatible with long-term soil health goals and improving the overall sustainability of agroecosystems. This Research Topic brings together 16 original research papers from a range of systems including crude oil-contaminated grasslands in semi-arid China, paddy fields in Vietnam, tallgrass prairie in Kansas, conventional and organic farms in New York State, and protected vegetable cultivation in Beijing. Together, they provide good evidence that the activity, composition, diversity, and network structure of soil microbial communities are key determinants of agronomic performance, and that our improved understanding is now providing new tools to promote agroecosystem multifunctionality and resilience.

## Disturbance can reduce soil functioning, however, amendments can help reverse this effect

Two of the papers demonstrate that contamination and salinity disrupt microbial function in ways that simple diversity metrics may miss. Wei et al. found that crude oil pollution of grassland soils increased bacterial and fungal diversity at moderate concentrations, however, ecosystem multifunctionality declined consistently as contamination increased. With respect to the practical application of phytoremediation approaches it suggests that cooperative microbial networks, which peaked at intermediate contamination levels, represent a window for intervention before competition and toxicity harm the community and reduce functioning. Malal et al. found a similar conclusion for salinity. In this study, they found a 20% or greater drop in soil multifunctionality associated with increasing NaCl stress, and the microbial networks that formed were denser but also less modular and therefore more fragile. In attempts to reverse this effect, they found that vermicompost addition partially restored diversity and stabilized those networks, while no positive response was observed for synthetic NPK fertilizer. In saline soils, which now affect an estimated 20% of irrigated agricultural land worldwide (Singh, 2021), it suggests that organic amendments may provide a ways to promote enhanced functional redundancy, particularly in comparison to inorganic nutrient addition. Zhao et al. studied the impact of resource-efficiency, finding that a composite microbial activator combining organic acids with phosphorus (P) mine tailings, an industrial waste product, increased bioavailable selenium (Se) by 56% while simultaneously raising soil available P by 21%, enriching Se-cycling bacteria in the process. Overall, the study shows that an industrial waste stream can be converted into a dual-purpose amendment addressing Se deficiency, while also improving P fertility. Thus, the approach may offer a strategy for promoting circular soil management while also addressing the ca. 1 billion people affected by Se deficiency (Genchi et al., 2023).

## Cropping decisions can be used to shape the soil microbiome

A series of papers in the Research Topic highlight that what farmers choose to grow, and the subsequent choice of management regime, leads to a restructuring of the soil microbiome. If managed correctly this can directly lead to improvements in crop yield, quality, and soil health. Shi et al. demonstrated that introducing legumes into a continuous potato rotation on the Loess Plateau shifted the rhizosphere community and increased commercial tuber yields by 28%, starch content by 35%, and vitamin C by 31%, while reducing the pathogen pressure associated with monocropping. Chen et al. found analogous benefits in sorghum systems in Guizhou Province, where introducing rapeseed into continuous monocultures suppressed pathogenic *Ascomycota*, elevated beneficial *Basidiomycota* and *Mortierellomycota*, and increased grain yield by 12% and 1,000-grain weight by 22%. These findings led the authors to call for greater promotion and introduction of crop rotation policies within the regional food supply chains. Zhou et al. showed that even within a single crop species, cultivar choice can lead to a restructuring of the rhizosphere microbiome. For example, across seven alfalfa cultivars, cultivar identity was found to explain 55% of the variation in bacterial community structure, with high-performing cultivars possessing denser and more cooperative microbial networks. This suggests that we need to consider extending breeding programs to target rhizosphere microbiome assembly alongside conventional above-ground agronomic traits. At the intercropping scale, Zhan et al. conducted a meta-analysis involving 323 studies and found that soybean intercropping consistently increases microbial alpha-diversity and yield, but that nitrogen (N) application above ca. 185 kg N ha^−1^ reduced these benefits, a threshold which has direct practical management relevance. In a more mechanistic study, Sun et al. tracked microbial communities across three ecological niches in a chili pepper-Chinese chives intercropping system. They found that nearly 70% of root bacteria in intercropped Chinese chives originated from pepper roots. This inter-host microbial transfer helps to explain how Chinese chives recruit beneficial *Bacillus* into their rhizosphere and why the combination may suppress soil-borne disease. Again, this provides direct biological evidence for the adoption of companion planting strategies to promote enhanced resilience in horticultural systems. In a complementary study, Son et al. scaled the comparison across 22 crops and multiple farms in New York State, finding that organic farming practice was a stronger determinant of rhizosphere community structure than plant species identity. They also used single-cell Raman spectroscopy to show that organic soils select for stress-resilient, lipid-accumulating microbial phenotypes which remain undetected by conventional sequencing approaches. Notably, manure-based inputs also increased the abundance of antibiotic resistance genes (ARG) within the core microbiome, highlighting a key trade-off whereby organic amendments may enhance soil function while simultaneously selecting for antimicrobial resistance determinants. This highlights the potential need to introduce systematic ARG monitoring in organic management systems. Finally, Pham et al. provided a fundamental taxonomic framework for paddy-system microbiome research by resolving three genera of nitrogen-fixing cyanobacteria in Vietnamese paddy soils and identifying genus-level morphological markers that enable classification without molecular tools. This has practical relevance for smallholder-managed paddy systems, where access to molecular sequencing technologies is limited.

## The future for agronomy is smarter amendments, not more inputs

Three of the papers in the Research Topic come to a similar conclusion that amendment strategy (the type and combination of inputs) has a much greater impact on the soil microbiome than the total input quantity. Further, they also found that the soil microbiome is a highly sensitive indicator of whether a strategy is working in terms of improving soil health. Xiao et al. showed that combining bio-organic fertilizer with nano-carbon in a coastal saline soil produced synergistic improvements in soil physical properties, bacterial diversity, and available nutrients that neither input achieved alone. They further showed that bacterial and fungal communities responded via distinct mechanistic pathways, highlighting that the need to study all parts of the microbiome, rather than focussing on bacteria alone. Notably, none of the studies considered the virome in soil which still represents an omission. Ma et al. demonstrated in cotton fields of the Yellow River Basin that substituting a fraction of synthetic N with an *Orychophragmus violaceus*-derived green manure, selected for its high C:N ratio, reduced total gaseous N emissions by more than 36%, reducing both NH_3_ volatilization and N_2_O emissions, while recruiting keystone taxa that promoted N immobilization. This is of significance given that agricultural N emissions are major contributors to both greenhouse gas forcing and reactive N pollution across terrestrial and aquatic systems. In tobacco cultivation, Liang et al. also found that substituting 50% of synthetic N with an organic-based fertilizer maximized yield, leaf quality, and microbial network integration simultaneously, while the 60% substitution level produced no additional benefit. The existence of a saturation threshold beyond which additional organic inputs no longer further restructure microbial communities or improve agronomic outcomes has direct implications for input-use efficiency, particularly in systems where organic fertilizers are associated with higher economic costs. Hu et al. also demonstrated that seed coating with a composite *Bacillus*-based PGPR formulation increased potato yield by ca. 39% over a chemical-only control across two field seasons, primarily by stimulating root biomass, promoting rhizosphere enzyme activity, and enhancing nutrient translocation to tubers. The better performance of the two-strain formulation relative to single-strain inoculants confirms that functional complementarity, integrating N-fixers, P-solubilizers, and phytohormone-producing taxa within a single seed treatment. This suggests commercial biostimulant products should take a consortia approach combining multiple desirable microbial traits.

## Tillage and land use shape microbial legacies detectable by sequencing

Two studies demonstrate that soil management history is reflected within the structure of the microbial community in ways that are not captured by traditional bulk chemical analyses. Yuan et al. showed that strip tillage, which disturbs only the planting row while leaving straw mulch between rows, increased microbial Shannon diversity relative to rotary tillage in the black soils of Northeast China. This effect persisted through winter freeze-thaw cycles in bacterial communities, although it was less pronounced in fungi. Cold-adapted indicator taxa associated with strip tillage were linked to the decomposition of crop residues and associated nutrient cycling processes, providing a mechanistic basis for the fertility benefits of undertaking conservation tillage in high-latitude systems where freeze-thaw dynamics strongly influence microbial community responses. In support of this, Banerjee et al. compared native tallgrass prairie with agriculturally managed soils in Kansas finding that land-use history, rather than contemporary P availability, was the primary determinant of microbial community composition. Overall, native grassland soils supported a higher bacterial biomass but a lower α-diversity than agricultural soils, which they attributed to the dominance of copiotrophic taxa under elevated labile C inputs in cultivated systems. Across sites, soil organic matter, moisture, and pH explained a large proportion of the variation in community structure and far more than the size of the plant-available nutrient pools. This reinforces the need to incorporate microbial responses to agricultural intensification into existing soil C models, enabling farmers to prioritize C dynamics alongside fertilizer inputs. Within native grasslands, microbial communities also retained signatures of differing fire frequencies and grazing regimes even in the absence of detectable differences in bulk soil chemistry, demonstrating that molecular sequencing can identify management legacies that conventional soil testing may miss.

## Insights and emerging research directions

**Figure 1:**
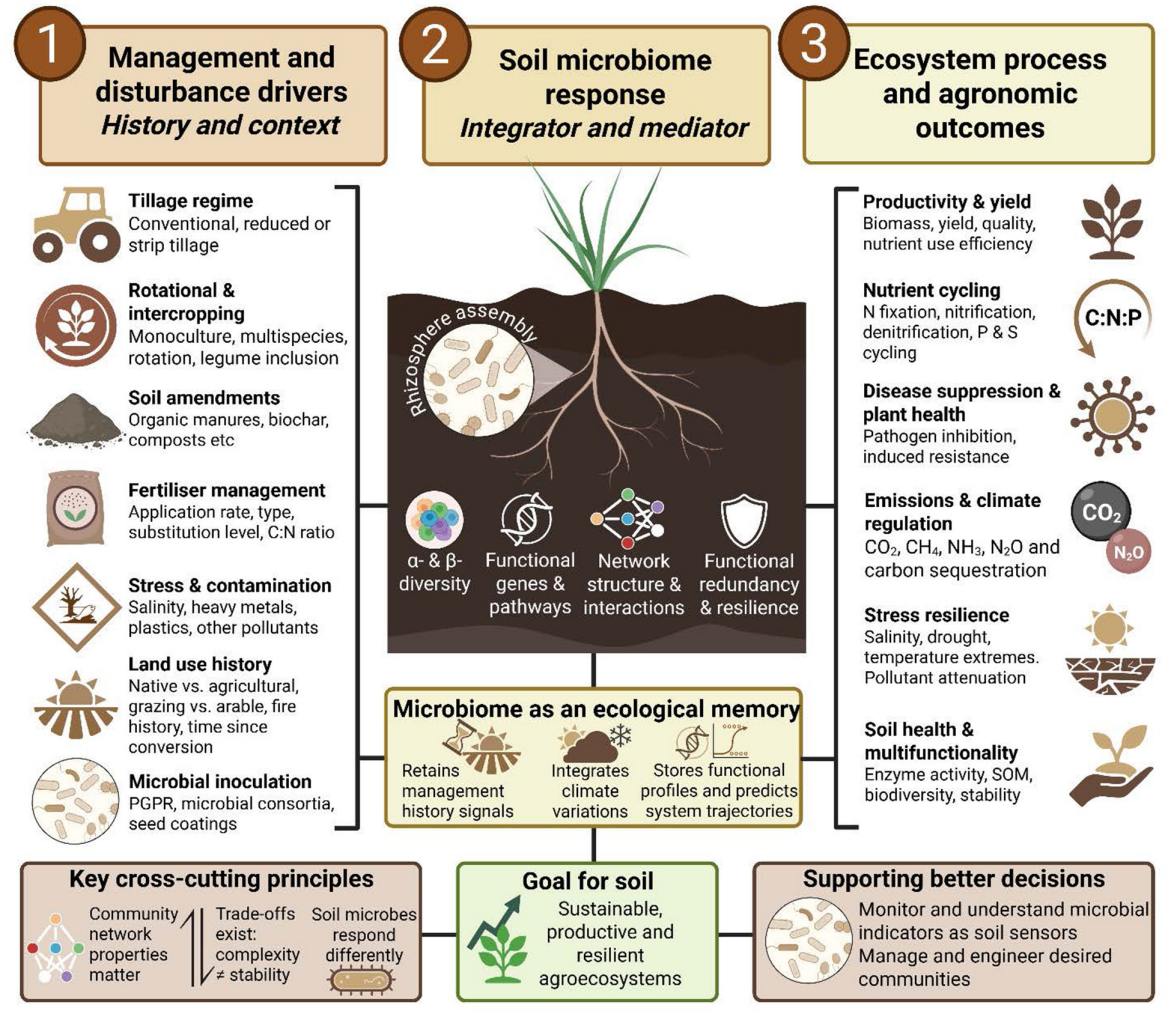
Schematic showing the soil microbiome as an integrator and senor of management history and a tool for agronomic improvement. Management and environmental drivers **(left)**, including tillage, rotation, amendments, fertilization, contamination, land-use history, and microbial inoculation, shape microbial community assembly, diversity, and network structure **(center)**. These community properties mediate key ecosystem processes and agronomic outcomes **(right)**, including crop productivity, nutrient cycling, disease suppression, emissions regulation, and soil health. Cross-cutting principles emerging from this Research Topic are summarized at the bottom, namely that (i) soil microbial network properties are important; (ii) bacteria, fungi, and archaeal communities may respond differently to the same pressures; (iii) intermediate diversity and input levels are often optimal; (iv) and trade-offs exist between network complexity and stability.

Taken together, these 16 papers identify several core principles that cut across systems and methodologies (Figure 1). Firstly, more diversity is not always better. The relationship between microbial diversity and soil function is non-linear, with intermediate diversity or intermediate amendment intensity producing better agronomic and environmental outcomes than either extreme. This challenges the conventional view of Delgado-Baquerizo et al. (2016) that a loss in microbial diversity will likely reduce multifunctionality, negatively impacting the delivery of a range of ecosystem services. Secondly, bacteria, fungi, and archaea respond differently to the same pressures, and studies that characterize only one kingdom risk drawing conclusions that are incomplete or actively misleading. We would also argue that more attention needs to be paid to the regulatory control of the microbiome by soil viruses, which are almost completely overlooked in most studies (Carreira et al., 2024). Thirdly, microbial co-occurrence networks, their complexity, modularity, and the ratio of cooperative to competitive links, represent sensitive integrators of soil community health that may be used to predict functional outcomes more reliably than taxonomic composition alone. Lastly, as highlighted in previous reviews (Maurya et al., 2020), the microbiome may be a better indicator of management history than bulk soil chemistry, capturing signals from tillage practice, stress events, and livestock grazing that conventional soil quality metrics do not.

In terms of the practical consequences for agroecosystem management, the interventions most associated with improved microbial community health were, well-balanced organic inputs with optimal C:N ratios, crop rotation with legumes, conservation tillage, and multi-strain biological inoculants. Importantly, these options are already available to most farmers worldwide. The primary constraint to their adoption is not the underlying science *per se*, but moreover the lack of translation, education and policy support mechanisms to promote their uptake. This should go alongside an improved mechanistic understanding of soil processes to ensure that the recommendations are maximized for different agronomic contexts. This will require a shift away from correlative inference to a causal understanding requiring confirmation of predicted microbial functions through enzyme assays and transcriptomic approaches, longitudinal tracking of community dynamics across multiple growing seasons, and evaluation under the heterogeneous and economically constrained conditions of real-world agricultural systems. The challenge now is for researchers to translate this foundational research into decision-relevant, context-specific guidance that can be widely adopted and operationalized by policymakers and the agricultural sector.

## References

[B1] Carreira C. Lønborg C. Acharya B. Aryal L. Buivydaite Z. Borim Corrêa F. . (2024). Integrating viruses into soil food web biogeochemistry. Nat. Microbiol. 9, 1918–1928. doi: 10.1038/s41564-024-01767-x 39095499

[B2] Delgado-Baquerizo M. Maestre F. T. Reich P. B. Jeffries T. C. Gaitan J. J. Encinar D. . (2016). Microbial diversity drives multifunctionality in terrestrial ecosystems. Nat. Commun. 7:10541. doi: 10.1038/ncomms10541 26817514 PMC4738359

[B3] Genchi G. Lauria G. Catalano A. Sinicropi M. S. Carocci A. (2023). Biological activity of selenium and its impact on human health. Int. J. Mol. Sci. 24:2633. doi: 10.3390/ijms24032633 36768955 PMC9917223

[B4] Maurya S. Abraham J. S. Somasundaram S. Toteja R. Gupta R. Makhija S. (2020). Indicators for assessment of soil quality: a mini-review. Environ. Monitor. Assess. 192:604. doi: 10.1007/s10661-020-08556-z 32857216

[B5] Singh A. (2021). Soil salinization management for sustainable development: a review. J. Environ. Manag. 277:111383. doi: 10.1016/j.jenvman.2020.11138 33035935

